# A Hypothesis for Bacteriophage DNA Packaging Motors

**DOI:** 10.3390/v2091821

**Published:** 2010-08-26

**Authors:** Philip Serwer

**Affiliations:** Department of Biochemistry, The University of Texas Health Science Center, San Antonio, Texas 78229-3900, USA; E-Mail: serwer@uthscsa.edu

**Keywords:** bacteriophage structure, biological energy transduction, biological signal noise, cryo-electron microscopy, single-molecule analysis

## Abstract

The hypothesis is presented that bacteriophage DNA packaging motors have a cycle comprised of bind/release thermal ratcheting with release-associated DNA pushing via ATP-dependent protein folding. The proposed protein folding occurs in crystallographically observed peptide segments that project into an axial channel of a protein 12-mer (connector) that serves, together with a coaxial ATPase multimer, as the entry portal. The proposed cycle begins when reverse thermal motion causes the connector’s peptide segments to signal the ATPase multimer to bind both ATP and the DNA molecule, thereby producing a dwell phase recently demonstrated by single-molecule procedures. The connector-associated peptide segments activate by transfer of energy from ATP during the dwell. The proposed function of connector/ATPase symmetry mismatches is to reduce thermal noise-induced signaling errors. After a dwell, ATP is cleaved and the DNA molecule released. The activated peptide segments push the released DNA molecule, thereby producing a burst phase recently shown to consist of four mini-bursts. The constraint of four mini-bursts is met by proposing that each mini-burst occurs via pushing by three of the 12 subunits of the connector. If all four mini-bursts occur, the cycle repeats. If the mini-bursts are not completed, a second cycle is superimposed on the first cycle. The existence of the second cycle is based on data recently obtained with bacteriophage T3. When both cycles stall, energy is diverted to expose the DNA molecule to maturation cleavage.

## 1. Introduction

**Figure 1.**
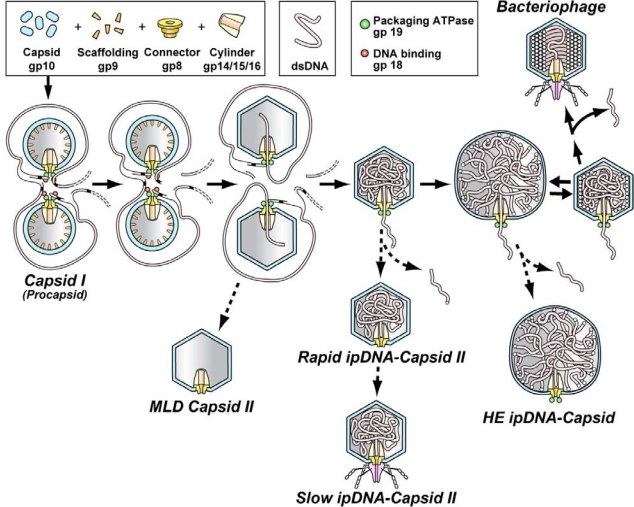
The DNA packaging pathway of the related bacteriophages T3 and T7 (adapted from [30]). The solid arrows indicate the proposed productive pathway in an infected cell. The dashed arrows indicate the pathways for generating the motor-related particles that have been observed by fractionation and characterization. Duplication of the early stages represents cooperativity detected by single-molecule fluorescence microscopy [93]. The legend at the top indicates the color-coding of both the DNA molecule and the various proteins.

Analysis of bacteriophage DNA packaging motors is performed to understand basic principles of energy transduction and associated signaling in multimolecular assemblies. A DNA packaging motor begins as a procapsid assembled from subunits in the absence of DNA (capsid I in the case of the related bacteriophages, T3 and T7; Figure 1). The motor binds a double-stranded DNA molecule and causes the DNA molecule to enter and package within the cavity of a symmetrical protein shell. This DNA packaging is an event that is accompanied by a change in the structure of the shell; this change usually includes increase in size (capsid II in Figure 1). The DNA molecule enters the cavity through an axial hole in two coaxial protein multimers that form a signaling center for the motor. The inner multimer is called the connector and is embedded in the capsid’s icosahedral shell at a five-fold rotational symmetry axis of the shell. The connector is made of 12 subunits, each a copy of a single protein, gp8 for T3/T7; the T3 and T7 proteins are named by gp, followed by the gene number from [1]. However, connector-like assemblies with other numbers of subunits are sometimes found when the connector protein is assembled outside of a bacteriophage particle. The monomer of the outer multimer is an ATPase [2–6].

The DNA packaging of some bacteriophages is accompanied by cleavage of the mature DNA genome from a multi-genome concatemer (maturation cleavage). A C-terminal endonuclease domain of the ATPase catalyzes the cleavage [2,3,5,7,8]. In the case of a concatemeric substrate for packaging, the DNA packaging ATPase (with its C-terminal endonuclease domain) is called either terminase (the nomenclature to be used here) or the large terminase protein. Terminase is accompanied by a smaller protein, sometimes called the small terminase protein, that binds DNA, is needed for initiation of packaging and sometimes has other activities (illustrated for T3 and T7 in Figure 1; gp18 is the small terminase protein; gp19 is terminase).

Bacteriophage DNA packaging motors are also models for understanding of both eukaryotic virus assembly and virus evolution, because DNA packaging motors evolved before the prokaryote/eukaryote splits. Specifically, herpes viruses also have a terminase and a connector [9,10]. The herpes virus terminase has sequence similarity to the phage terminases [11–13]. Thus, the connector and terminase proteins had evolved by about 1.6 billion years ago [14]. The high speed and low cost of bacteriophage propagation have been a foundation for relatively thorough genetic/biochemical/biophysical analysis of bacteriophage DNA packaging motors. *In vitro* systems have, for example, shown that DNA with a previously introduced single-stranded break (nick) in the phosphodiester backbone is packaged in the case of both ϕ29 [15,16] and T4 [17]. A nick in a comparatively short packaging substrate (<200 base pairs) did, however, partially inhibit T4 packaging, primarily by slowing it (Figure 5d in [17]). Thus, if transmission of torque is a required aspect of packaging, the protein component of the motor must resist DNA rotation.

The analysis of bacteriophage DNA packaging motors has included the testing of proposed motor mechanisms by single-motor, real time, visible light-based nanometry and fluorescence microscopy of *in vitro* DNA packaging in systems of purified components. Several proposed motor mechanisms require rotation of the connector [18–22]. However, no rotation of the bacteriophage ϕ29 connector was found (with probability >99%), when nanometry was used to measure packaging progression and single-motor fluorescence anisotropy was used to measure packaging-associated connector rotation [23]. The conclusion of no motor-associated connector rotation had previously been drawn in the case of bacteriophage T4 when packaging continued after the potential for connector rotation was removed by genetically modifying the connector subunits [24].

Among the various previously proposed motor mechanisms, some aspects of a cycle based on thermal ratcheting [25,26] did survive the above tests. The definition used here for thermal ratcheting is external force-dependent rectification of thermal motion (see also [27,28]). At the earliest stage of packaging, one proposed rectifier was intracellular osmotic pressure; a second was motor/DNA binding initiated by reverse DNA motion [26]. Other investigators have subsequently adopted motor/DNA binding-based thermal ratcheting, at least in its most general form [23,29]. However, details of a hypothesized thermal ratchet have not been proposed with cognizance of data obtained after 2003.

In past studies of these details, most discussion has included the implicit assumption that each DNA packaging motor has a cycle of one type. However, isolation of motor-related particles from bacteriophage T3-infected cells revealed capsid hyper-expansion that suggested adding to the cycle previously investigated (type 1 cycle) of a second cycle (type 2 cycle) at the later stages of packaging, at least *in vivo*. The proposed type 2 cycle includes ATP-driven hyper-expansion and contraction of the shell [30].

Although shell hyper-expansion has not been detected for other bacteriophages, some data suggest this possibility. First, a sudden decrease in resistance to *in vitro* bacteriophage λ packaging has been observed at 90% packaging by single-molecule nanometry [31]. This decrease is possibly caused by shell hyper-expansion, although capsid rupture is the explanation proposed by the authors. Second, stabilization of a hyper-expanded λ capsid is a possible explanation for the recent observation that a capsid-stabilizing, decoration protein of the λ capsid (D protein) is required not only for DNA retention after packaging, but also for packaging of the last 15–20% of the λ genome [32].

A possibly related phenomenon is that the shell subunits of some bacteriophages, such as HK97, are covalently joined to each other by chain-like cross-links established during assembly [33,34]. Assuming that the HK97 covalent cross-links are produced before packaging is completed, these cross-links could have evolved to stabilize a hyper-expanded capsid at the end of DNA packaging. In this case, any change in cross-linked shell size would occur by refolding of each of the subunits, possibly via a rubber-like (and, by analogy, exothermic) stretching. The cross-links prevent hyper-expansion via inter-subunit translation and rotation. In confirmation, a recent study reveals that the procapsid-to-mature capsid transition for HK97 (equivalent to the capsid I-to-capsid II transition for T3; Figure 1) occurs via change in protein folding [34]. This change is exothermic for bacteriophage P22 [35]. Given the structural analogy of the λ D protein and the HK97 cross-links [36], at least some functions are likely to be the same for these two aspects of bacteriophage capsids.

The objective of the current study is to derive a type 1 cycle that explains all current data and that provides means for initiating a type 2 cycle. To help compare studies of the various bacteriophages, the extent of packaging will be quantified by the ratio (*F*) of the length of DNA packaged to the mature genome length.

In deriving the type 1 cycle proposed here, the assumption is made that the basics of the type 1 cycle are the same for all bacteriophage DNA packaging motors. This assumption is confirmed by the structure-based similarity of the ϕ29, P22, SPP1 and T7 connectors, without detected amino acid sequence similarity [37,38], and the sequence-based similarity of all terminases [7,11,12]. The N-terminal region of terminases has even been aligned with the DNA packaging ATPase of bacteriophage ϕ29 [39]. Bacteriophage ϕ29 has a monomeric DNA packaging substrate and has a packaging ATPase with no known endonuclease activity [6,41]. The proposed cycle has aspects that should be applicable to all double-stranded DNA viruses, including those of significance for both medicine and ecology. In addition, some aspects should be applicable to energy transducing systems of other types, including systems in which protein folding is assisted by chaperonins.

## 2. Examination of some past assumptions

As reviewed in [5,6], most of the (type 1) cycles previously proposed for DNA packaging motors were based on the assumption that the ATP usage per base pair packaged is constrained to be uniform throughout packaging (constant ATP assumption) and is equal to 0.5 ATP cleavages per base pair packaged. The sources of the constant ATP assumption are chromatographic measurements of the number of ATP molecules used per base pair packaged during unsynchronized *in vitro* packaging in the case of ϕ29 [41] and T3 [42]. But, the data of the latter two studies revealed ATP usage averaged over all values of *F*, because the packaging was not synchronized. The ATP usage per base pair packaged has never been measured as a function of *F*, to the author’s knowledge. Performing this measurement is a goal for the future, possibly achievable by single-molecule analysis because single ATP cleavages have been detected by use of single-molecule fluorescence microscopy ([43,44], for example). The constant ATP assumption cannot be rationalized by analogy to non-viral eukaryotic motors because DNA packaging motors evolved before non-viral eukaryotic motors and are not under the same constraints, as further discussed in Section 7.

In addition, the constant ATP assumption implies usage of ATP that is less efficient than it would be if the ATP used per base pair packaged increased as *F* increased, based on two lines of evidence. First, energetics-based computer simulations of packaging dynamics for bacteriophage ε15 revealed “negligible” (<2 pN) requirement for force when *F* was less than 0.4 [45]. These simulations have been accurate in the past; they correctly predicted the randomness of newly packaged DNA conformation, for example [46]. Second, single-motor nanometry-based determination of packaging forces for bacteriophages ϕ29 [47,48], λ [31] and T4 [49] had previously yielded basically the same conclusion. Given that the osmotic pressure of the cytoplasm of *Escherichia coli* is about 5 atmospheres [50], an ATP-independent, osmotically derived packaging force of about 6 pN would exist *in vivo*, independent of ATP consumption, if the capsid interior were empty. That is to say, when packaging occurs *in vivo*, ATP-derived energy is possibly not needed to package until *F* = ∼0.3 [48].

Both the simulations and the nanometry also agree that, as *F* increases, the force required for packaging is a steeply increasing function of *F* and can be as high as 125 pN at the end of packaging in the case of the ε15 simulations, and 50–70 pN in the case of the nanometry; the capsid is assumed constant in size. The power delivered by the ϕ29 motor decreases progressively to zero *in vitro* at the lower *F* values [48]. Thus, the constant ATP assumption implies that roughly half of the ATP to be cleaved is wasted before it is even needed. Viewed in the context of evolution, the constant ATP assumption produces an outcome likely to be subject to negative selection. The result of negative selection can be either *F*-dependence of the ATP utilization in the type 1 cycle or introduction of the type 2 cycle (or both). The type 1 cycle might not even begin *in vivo* until roughly one third of the DNA was packaged. To the author’s knowledge, nanometry-based tests have not yet been made of the effect of osmotic pressure gradients on the *in vitro* packaging of a bacteriophage DNA packaging motor. The type 1 cycle proposed here does not depend on the constant ATP assumption.

Although a previous manuscript [26] does propose two cycles and does propose ATP usage (per base pair packaged) that increases as *F* increases, the detailed mechanisms proposed have other aspects that are in conflict with more recently obtained data. First, the small terminase subunit was attached to terminase and was the component of the motor that bound the DNA molecule during cycling. However, the small terminase is not part of the ϕ29 motor because ϕ29 does not have this protein. Yet, the ϕ29 connector has structure-based similarity to the connectors of terminase-dependent bacteriophages. The ϕ29 packaging ATPase has sequence-based similarity to the terminases. Furthermore, the T4 motor does not need the small terminase protein after initiation of DNA packaging [51]. Thus, the DNA packaging ATPase (gp19 for T3; Figure 1) is assumed here to act without its smaller companion. In addition, several recent studies (described below) have produced data that indicate the need for further modification of the type 1 cycle proposed in reference 26. These data will form the basis for the proposal of a type 1 cycle revised in some, but not all, aspects. The type 2 cycle will be subjected to only limited discussion here.

## 3. Key data from previous studies of the type 1 cycle

The key data discussed in this section will be used as the basis for the type 1 cycle to be proposed. Of course, other data exist. The author is not aware of any other data with which the type 1 cycle presented below is in conflict.

### 3.1. Thermal ratcheting and its limitations

An aspect of the type 1 cycle to be proposed is that this cycle includes a time period in which the DNA molecule does not move relative to the connector/ATPase multimer because the DNA molecule is bound to the ATPase multimer. This period of no DNA motion will be called a dwell, as proposed in [52]. The DNA molecule is packaged between dwells in a period called a burst. The evidence for dwells and bursts was the *F vs.* time relationship observed by high-resolution single-molecule nanometry of *in vitro* ϕ29 DNA packaging [52]. The DNA molecule was under constant optical trap-maintained force of 8 pN, equal to the DNA packaging force needed when *F*∼ = 0.38 [48]. In the type 1 cycle to be proposed, both a dwell and a burst will occur.

Details of the nanometry provided additional information. The dwell time decreased with increase in [ATP], although the burst time was independent of [ATP]. This observation was interpreted to mean that binding of ATP caused the dwell (*i.e.*, caused DNA binding) and that the burst occurred in-between Details of the nanometry provided additional information. The dwell time decreased with increase in [ATP], although the burst time was independent of [ATP]. This observation was interpreted to mean that binding of ATP caused the dwell (*i.e.*, caused DNA binding) and that the burst occurred in-between ATP binding-associated dwells [52]. However, this observation also implies that the bound ATP caused a change that occurred during the dwell and that increased in magnitude as the number of bound ATP molecules increased. Among the changes observed after ATP binding is decrease in the Gibbs free energy (tighter binding) of the bound ATP [52,53]. In the type 1 motor mechanism to be proposed, this decrease will be essential to activation of a component of the connector. Nonetheless, the ATP binding site is assumed to be on the ATPase (terminase) because, in the case of bacteriophage T3, a strong ATP binding site has been found on the T3 terminase, gp19, although no ATP binding site was observed on the connector, gp8 [42]. DNA binding-associated dwell-periods were also an aspect of the proposed thermal ratchet-based type 1 cycle of [26]. The thermal ratchet was biased by force from an osmotic pressure differential across the capsid’s shell (higher outside) and possibly a motor-derived, oscillating electrical field that generated net force on the DNA molecule.

A purely thermal ratchet-based motor mechanism is now in conflict with the following subsequent observation also made by high-resolution nanometry of the ϕ29 DNA packaging motor. As the optical trap-derived force opposing packaging increased at a fixed *F* for packaging slowed by the methylation of a patch of 10 DNA phosphates, the time taken to package this patch was reduced by an amount too low by over four orders of magnitude to be explained by diffusion only [16]. That is to say, the DNA movement of the burst was caused by forward-directed force (*i.e.*, a force in the direction of packaging) and was not generated by passive diffusion. This force could not have had a significant component from osmotic pressure, based on the composition of the *in vitro* packaging mixture, which had no compound added to mimic the intracellular water activity. Thus, the motor has to be more than either a purely thermal ratchet or a purely osmotic pressure-biased thermal ratchet. Nonetheless, an osmotic pressure gradient is likely to provide some of the DNA-driving force *in vivo* and also in the case of *in vitro* systems that depend on the presence of polymer, such as those for P22 [54], T7 [55], T3 [56], SPP1 [57] and T4 in a recently developed system [17].

A related aspect of the type 1 cycle is coordination among ATPase multimer subunits in producing ATP binding-induced DNA binding. This coordination was deduced in the case of the ϕ29 DNA packaging motor from the sharpness of a plot of the frequency of any given dwell time *vs.* dwell time. The sharpness of this plot implied a minimum of 2 ATP binding events to generate a dwell [52]. The ATPase multimer was five-membered during packaging in this system [21,58]. One possible mechanism for coordination is ATP binding-induced contraction of a ring of ATPase molecules. The result would be DNA binding via steric clamping by the ATPase ring of the DNA molecule being packaged. Steric clamping will be a non-essential part of the mechanism proposed here (clamping was originally suggested to the author by S. C. Hardies). Other modes of binding are also consistent with the data, although no consensus DNA binding site has yet been reported in the N-terminal ATPase domain (motor domain) of terminases.

### 3.2. Connector dynamics in the type 1 cycle

Another aspect of the proposed type 1 cycle is that the connector has two essential functions, transmission of information and transduction of force. This aspect is based on the following observations of the bacteriophage SPP1 connector. (1) The DNA channel of the SPP1 connector is narrow enough to contact a DNA molecule in the channel. The channel is ∼18.1 Å in diameter at its narrowest point, as found by obtaining the x-ray crystallographic structure of a connector protein 13-mer and extrapolating the structure of the 13-mer to the 12-mer present in bacteriophage particles [38]. This diameter is, in fact, about 20% smaller than the diameter of the narrowest channel that could contain a DNA molecule without contact with the connector. (2) Blockage of packaging occurred when both intra- and inter-connector subunit motion was inhibited by disulfide cross-linking of connector subunits to each other; reversing the cross-links restored the packaging activity [59]. (3) Some point mutations of the SPP1 connector protein either abolished or decreased both DNA packaging and the ATPase activity of the packaging ATPase without disrupting assembly of the connector in the SPP1 procapsid [38,60].

The conclusion drawn was that DNA packaging-associated “cross-talk” existed between the connector and the packaging ATPase and that the connector was part of the DNA packaging motor [38,60]. Assumption of this cross-talk is supported (although not proven) by the observation that the rate of ATP binding to the ϕ29 motor is independent of the nanometry-determined force on the motor [53,61], as though the force is sensed by one motor component and the ATP binding is to another. In the type 1 cycle to be proposed, the force sensor will be the connector; the ATP binding element will be the ATPase multimer. An explanation will be provided for evolution of the partitioning of these functions to two proteins. Both functions are in a single protein in the case of non-viral eukaryotic motors.

The proposed details for the above activities of the connector include change in conformation of peptide segments of the connector subunits. This aspect is based, first, on the analytical calculation that peptide segments have the capacity for achieving up to 4.7 different conformations for which neither stability nor activation energy is comparable to the energy of thermal motion [62]. This number is approximately the number of conformations that can be an independent part of a type 1 cycle of a DNA packaging motor. The value, 4.7, depends primarily on the number of different amino acids (twenty) and is independent of peptide chain length. The conformational mobility aspect is also based on the finding of regions of assembled connector proteins that are channel-proximal and that are likely to be conformationally mobile (to be called mobile peptide segments), in that they are without regular secondary structure.

**Figure 2.**
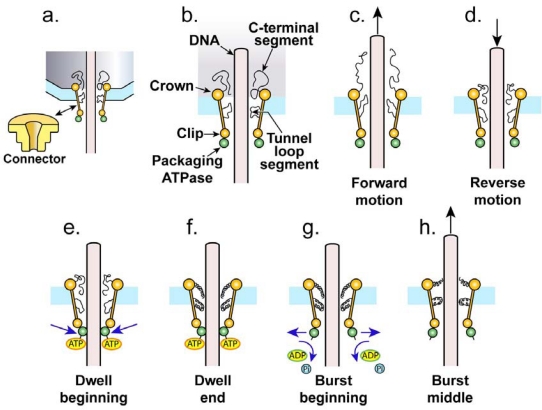
The components of the proposed type 1 cycle, side view. To simplify the drawing, the correct symmetry of the ATPase multimer, assuming it to be five-fold, is not represented. **(a)** The connector (yellow) is shown as represented in Figure 1 (lower left, in isolation) and also at higher resolution while embedded in the shell (light blue) with terminase (green) attached to it. **(b)** The components of the signaling center of the motor are shown at higher magnification with the labeling used in the text. **(c)** The motor is shown at the beginning of a cycle with DNA molecule undergoing forward thermal motion, *i.e.*, thermal motion into the cavity of the shell. **(d)** The motor is shown with DNA molecule undergoing reverse thermal motion. **(e)** The motor is shown at the beginning of a dwell, with ATP (yellow oval with orange border) bound to terminase and terminase bound to DNA molecule. **(f)** The motor is shown at the end of a dwell with mobile peptide segments activated; coiling illustrates activation, but the true activated conformation is not known. **(g)** The motor is shown at the beginning of a burst, with terminase no longer binding the DNA molecule, ATP cleaved and mobile peptide segments pushing on the DNA molecule as they begin to deactivate. **(h)** The motor is shown in the middle of a burst. As the burst proceeds, the motor returns to its state in (c, d).

The connector has subunits each of which has two mobile peptide segments by these criteria. To illustrate the mobile peptide segments in relation to the rest of the motor, Figure 2a shows the DNA molecule (tan), connector (yellow), packaging ATPase (green) and outer shell (blue). The first of these two peptide segments is a C-terminal, 40 residue peptide segment without a unique structure (disordered), seen next to the widest end (crown) of the SPP1 connector, inside of the outer shell (Figure 2b) [38]. In the smaller ϕ29 connector, this disordered peptide segment is 24 residues long and encompasses the region that corresponds to the crown of SPP1 [22,38]. The second of these two peptide segments forms a loop in the SPP1 connector (tunnel loop) that is attached at both ends to an α-helix and projects into the axial channel at the smallest diameter of the channel (∼18.1 Å) (Figure 2b) [38].

For the tunnel loop, genetic analysis supports a function in SPP1 DNA packaging. Among SPP1 connector-associated packaging mutations, five were in the tunnel loop region; four of the 15 amino acids in the SPP1 tunnel loop were mutated. One of these mutations reduced both the efficiency of packaging and the activity of the SPP1 packaging ATPase [38]. The tunnel loop is disordered in the ϕ29 [21,22], but not the SPP1 [38], connector. The tunnel loops of the 12 SPP1 connector subunits can simultaneously all engage the major groove of the DNA double helix, but they must be translated relative to each other along the connector axis because of the tightness of fit [38]. In the proposed type 1 cycle, the conformational changes of the mobile peptide segments have two functions, motion sensing/signal initiation and force transduction. Both C-terminal and tunnel loop segments are assumed to be participants, although the evidence for the tunnel loop is more complete in the case of SPP1. In the case of ϕ29, the tunnel loop may be redundant at the earlier stages of packaging because a preliminary study [63] has revealed that deletion of the tunnel loop inhibits packaging only near the end.

Lebedev *et al.* [38] have already proposed that engagement of tunnel loops with the major groove of the DNA double helix is either signaling or force-delivering in character. In the latter case, the proposed type 1 cycle is a high-detail version of the rotating connector-driven forward motion originally proposed [18], but with the DNA molecule rotating relative to an immobile connector, not the connector rotating relative to the outer shell and the DNA molecule. Rotating the DNA molecule relative to an immobile connector has the disadvantage that supercoils will be introduced in the DNA as it is packaged. In addition, this mechanism is unlikely, based on the observed packaging of mismatched regions in the case of ϕ29 [16] and T4 [17] and unpaired single-stranded regions in the case of λ [64], as further discussed below. The type 1 cycle proposed here does not have these conflicts with the data.

### 3.3. Fine structure of the single-molecule nanometry

Finally, the type 1 cycle proposed here was developed with the constraint that it must explain the recently observed fine structure of the *F vs.* time relationship obtained by high-resolution, single-molecule ϕ29 nanometry. When observed at high resolution and force also relatively high, 40 pN, dwells were separated not by a single burst, but by a series of four “mini-bursts”. The DNA molecule moved 2.5 base pairs per mini-burst [52]. The non-integral DNA progression during mini-bursts has been a mystery. The type 1 cycle proposed here explains the number of these mini-bursts and their production of non-integral DNA progression during packaging.

## 4. The proposed type 1 cycle

The type 1 cycle proposed here begins with the connector’s mobile peptide segments in contact with the DNA molecule being packaged. The axial channel is so small at its narrowest point (∼18 Å in diameter at the tunnel loops, as derived from packaging non-active particles in [38]) that the mobile peptide segments (C-terminal and tunnel loop) are not in a unique relationship to the DNA molecule, although they contact the DNA molecule. The data are not yet sufficient to propose the details for the initiation of packaging, *i.e.*, the events that occur before the state of Figure 2a, b is achieved.

### 4.1. The ratcheting component

As previously proposed [26], the DNA molecule now moves by diffusion, either into (forward) or out of (reverse) the capsid. An osmotic pressure gradient biases diffusion in the forward direction, as supported by the promotion of DNA packaging in some *in vitro* systems by neutral polymers (discussed above). This polymer-enhancement of DNA packaging is an osmotic pressure-generated, not an excluded volume-generated, effect [65]. Forward motion causes stretching of the mobile peptide segments in the forward direction (Figure 2c). As discussed above, the osmotic pressure present *in vivo* is sufficient so that the type 1 cycle need not proceed further until *F* = ∼0.3.

Eventually, reverse diffusion occurs (Figure 2d). The probability and magnitude of reverse diffusion increase as obstacles to packaging increase. These obstacles include steric and charge-charge repulsive interactions of both packaged DNA segments (reviewed in [4]) and comparatively small molecules accidentally packaged. One of the proposed purposes of the mobile peptide segments is to sense this reverse motion by changing conformation (Figure 2d). This changed conformation initiates a signal that is part of the connector/ATPase cross-talk previously observed for bacteriophage SPP1 [38]. This signal is transmitted through the connector to the packaging ATPase (terminase) multimer.

The type 1 cycle continues with a dwell-response to the signal sent to the ATPase multimer from the connector. The dwell-response begins with increase in ATP binding by the ATPase subunits. As shown in Figure 2e, ATP binding to the ATPase eventually results in ATPase/DNA binding that stops the reverse motion and produces a dwell. The DNA binding possibly, but not necessarily, occurs by clamping. During the period of the dwell, the mobile peptide segments are held in a conformation (Figure 2e) previously generated by strain during reverse motion of the DNA molecule (Figure 2d). The conformation of mobile peptide segments cannot return to the original because forward DNA motion is prevented and the unoccupied space in the channel is insufficient. The DNA molecule may also be under compressive strain from ATPase-DNA binding during reverse DNA motion and trapping at the other end by the mobile peptide segments (Figure 2e). Analysis of connector-proximal DNA (by Förster resonance energy transfer between dyes at two places on the DNA molecule) has revealed DNA compression during *in vitro* T4 DNA packaging stalled from outside of the capsid [51]. However, the interpretation of this latter study was compression via a power stroke of the ATPase. A power stroke of the ATPase does not occur in the type 1 cycle proposed here. Thus far, the type 1 cycle proposed here is not distinguishable from the cycle of a (power stroke-free) thermal ratchet.

### 4.2. The non-ratcheting component: ATP-driven mobile peptide segment activation

After the start of the dwell, the proposed type 1 cycle differs from the cycle of a thermal ratchet in that the connector’s mobile peptide segments are activated as the dwell continues (Figure 2f; activation is illustrated by coiling of the mobile peptide segments). After the dwell starts and before activation, the activation is made energetically possible by lowering of the Gibbs free energy of the bound ATP molecules. The dwell provides time for both this lowering and the linked, seesaw-like raising of the Gibbs free energy of the mobile peptide segments. The activation occurs while the ATP is bound, not while (or after) the ATP is cleaved. The activated, connector-associated mobile peptide segments have conformations not accessible in the absence of the pathway for Figure 2,c–f. In support of this sequence of events, non-sigmoidal (non-cooperative) [ATP]-dependence of both ϕ29 packaging velocity [53] and mean ϕ29 dwell time [52] has led to the conclusion that an initial ATP binding is followed by tightening of ATP binding, thereby short-circuiting the cooperativity otherwise expected [52]. The mobile peptide segments, as drawn in Figure 2, are schematic illustrations and are not accurate representations of conformation.

The apparent paradox of energetic linkage via DNA “stillness” is resolved by the following. Although the DNA molecule does not move relative to the motor, movements within the ATPase and connector subunits still occur. ATPase/connector cross-talk via these movements is physically realistic because movements of this type have already been shown to occur in the case of ABC transporters. In the case of the ABC transporters, movements within dimeric ATP binding domains occur after ATP binding and before ATP cleavage, as proposed above. The movements of the ATP-binding domains cause movements in transport-generating, α-helix-rich transmembrane domains that sometimes, like the connector subunits of bacteriophage DNA packaging motors, are in a separate protein [66,67]. Thermal oscillations potentially assist transfer of energy from ATPase (terminase)-bound ATP to the connector-associated mobile peptide segments. Sufficient information does not exist to propose either complete detail for ABC transporters [66,67] or any details for bacteriophage DNA packaging motors. The process involved for DNA packaging motors may be analogous to protein folding promoted by immobilization in the internal cavity of a chaperonin complex, a process that sometimes occurs at the expense of ATP binding energy [68–70].

**Figure 3.**
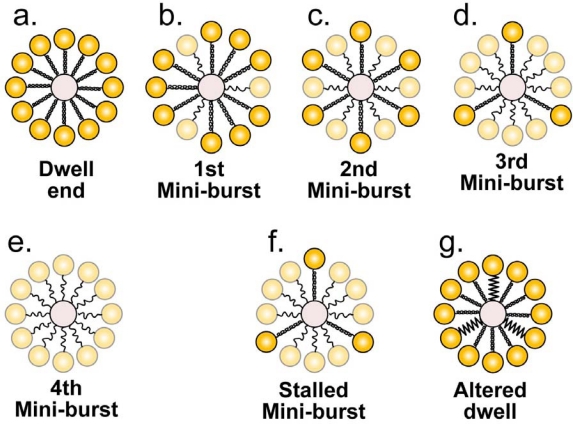
A transverse section-based representation of the firing of the connector subunits. A coiled peptide segment and a dark sphere represent an activated connector subunit. An uncoiled peptide segment and a light sphere represent a completely deactivated connector subunit. The DNA molecule is in the middle. This representation of the connector is a section with some details missing and modified to avoid confusion. Specifically, the clip and crown are, together, represented by one circle and only one peptide segment is shown; the subunits are not in contact. **(a)** The motor is shown at the end of a dwell with all 12 subunits activated. The motor is shown at the end of the **(b)** first, **(c)** second, **(d)** third and **(e)** fourth mini-bursts. **(f)** The motor is shown during a stalled mini-burst. **(g)** The motor is shown after an activation of the mobile peptide segments that began after a stalled mini-burst.

The lowering of the Gibbs free energy of ATPase-bound ATP signals the ATPase multimer to cleave the ATP molecules and, thereby, to release (perhaps unclamp) the DNA molecule and start a burst (Figure 2g). During the burst, the connector’s activated mobile peptide segments deactivate while pushing the DNA molecule. To illustrate additional details of the deactivation/pushing, Figure 3 begins by schematically illustrating the end of the dwell in projection along the DNA axis (Figure 3a). Each coiled fiber represents both activated C-terminal and activated tunnel loop segments of one connector subunit; each dark yellow ball represents (schematically) the rest of a connector subunit. For clarity, the drawings of Figure 3 are not true projections (see legend to Figure 3).

The following aspect of connector-based energy transduction directly satisfies the empirically derived [16] constraint of four mini-bursts per burst. The connector’s 12 subunits act sequentially in four groups of three subunits. That is to say, one group of three subunits initially pushes the DNA molecule (fires, in the terminology of an internal combustion engine) and is followed by the other three groups, one firing after the other (Figure 3,b–e). Firing is represented in Figure 3 by lightening of a yellow ball and uncoiling of the attached fiber. To reduce the tightness of fit in the channel [38], (1) the unfired subunits of Figure 3 rotate at the clip so that their crowns are further from the channel’s axis (and away from each other) than crowns of fired subunits and (2) after firing and uncoiling, mobile peptide segments move away from the channel. This latter movement is not represented in Figure 3. The physical basis for firing in groups of three is, therefore, proposed to be steric constraint on the number of mobile peptide segments that fit in the channel together with the DNA molecule.

In addition to explaining the results of nanometry and making room for the mobile peptide segments, firing in four groups of three keeps the DNA molecule centered. Unlike firing in four groups of three, firing in six groups of two would not avoid thermal motion-generated de-centering. The remaining alternative is firing in three groups of four, which would keep the DNA molecule centered, but might cause problems with tightness of fit.

In Figure 3, the conjecture is made that the three firing subunits of a mini-burst are symmetrically located, even though not linked to each other and restricted to thermal motion before firing begins. This conjecture has some basis (but, is not proven) from analysis of electrical systems in that promotion of symmetrical response via combination of more than one independent noise patterns has been found in theory [71].

A type 1 cycle must account for the observations that (1) perturbations of DNA structure do not necessarily inhibit the pushing of the DNA molecule and (2) the number of base pairs (2.5) packaged per mini-burst is non-integral [16]. The perturbations include mismatched, double-stranded regions (ϕ29: 10 bases [16]; T4: 10 bases [17]) and unpaired single-stranded regions (λ: 19 bases [64]). These observations conclusively demonstrate that recognition of a unique DNA secondary structure is not necessary. (They also indicate that firing can occur less often than one would calculate from the average ATP usage per base pair.) Thus, the firing of the connector subunits is proposed to push the DNA molecule via non-specific forward sweeping of the DNA molecule by the mobile peptide segments. The sweeping is accomplished by steric interaction (*i.e.*, contact with the DNA molecule is involved) and possibly also hydrodynamic interaction of the activated mobile peptide segments with the DNA molecule. No structure-specific engaging of a DNA component, such as the major or minor groove, occurs in the proposed type 1 cycle.

Sweeping drives the DNA molecule forward until either (1) the activation energy for all 12 connector subunits is dissipated and sweeping stops (Figure 3e) or (2) the motor has to work against a force that is high enough so that the sweeping is not completed and the DNA molecule undergoes reverse thermal motion before the final sweep (mini-burst) is completed (stall; Figure 3f). A stall arises from resistance to packaging produced both by the DNA segments already packaged and by small molecules (RNAs and proteins, for example) that have been accidentally packaged in an infected cell. Thus, the proposed type 1 cycle will not stall as often (or maybe not at all) *in vitro*, unless the macromolecular composition of the cellular cytoplasm is mimicked.

The reverse thermal motion of the stall of Figure 3f initiates a type 2 cycle via the following proposed mechanism. This reverse DNA motion causes a dwell and, then, refolding of the mobile peptide segments, as it did at the beginning of the type 1 cycle. But, the mobile peptide segments from only nine subunits have completed firing. Thus, the dwell is altered in that only nine mobile peptide segments refold to the conformation of Figures 2f and 3a. The remaining three mobile peptide segments have not completed firing and are in a space larger than experienced in an unaltered type 1 cycle. Therefore, these three mobile peptide segments refold to an altered, although activated conformation (Figure 3g) while the DNA molecule is bound to the ATPase multimer and the Gibbs free energy of the bound ATP is lowered. As in a type 1 cycle with an unaltered dwell, the next event is ATP cleavage, followed by a burst. However, the altered activated conformation causes an altered burst. The altered burst includes movements of the connector that initiate capsid hyper-expansion and, then, the rest of the type 2 cycle. The author reserves proposing further details until more information is obtained about the type 2 cycle, while noting that an empirical precedent exists in that the bacteriophage T4 connector initiates a capsid expansion roughly equivalent to the expansion that occurs during the capsid I to capsid II transition in Figure 1 [72].

## 5. The type 2 cycle and terminase cleavage of a concatemer

The details of the type 2 cycle are also not proposed here beyond what has previously been proposed in [26]. The proposed interaction of the two cycles is the following. (1) The type 2 cycle restarts the type 1 cycle by hyper-expansion-generated reduction of the concentration of packaged DNA segments and pumping-generated removal of the accidentally packaged small molecules from the DNA-containing cavity of the capsid. (2) The pumping works by expansion/contraction, coupled with changes in permeability, of the shell. (3) The type 2 cycle stops when not triggered by the type 1 cycle. (4) The type 1 cycle continues running and re-triggers the type 2 cycle when the type 1 cycle again stalls. (5) Eventually, both cycles undergo a stall (called a co-stall). In the case of T3 DNA packaging *in vivo*, the first type 2 cycle occurs at *F* ∼ 0.28 [30]; in the case of λ DNA packaging *in vitro*, the *F* value at packaging force reduction suggests that the only type 2 cycle occurs at *F* ∼ 0.9 [31].

The hypothesis presented here is extended to propose that a co-stall initiates the maturation cleavage of a concatemer (Figure 1). The proposed mechanism is alternative channeling of the energy of bound ATP, since this energy can no longer be channeled to DNA packaging. Specifically, as the time of co-stalling increases, the probability increases that the energy of bound ATP is alternatively channeled to expose the DNA molecule to the endonuclease domain of terminase. When that happens, the maturation DNA cleavage occurs. Sufficient information does not exist to propose further details. By this proposal, the probability of maturation cleavage is never zero at any stage of packaging. Thus, a background of erroneous maturation cleavages occurs and erroneous cleavages are made more frequent by a dwell or a stall during the normal cycling of the motor.

In support of these proposals, observation has been made of prematurely cleaved, incompletely packaged T3 genomes in capsids obtained from bacteriophage T3-infected cells [30,46] (see Figure 1). Based on the lengths of the cleavage products, cleavage positions are sometimes quantized, as though occurring during the type 1 cycle stalls that trigger the type 2 cycle [30]. In further support of the proposed cleavage-promoting alternative pathway, artificial slowing of *in vitro* T3 packaging accelerates the maturation cleavage of concatemers [73]. Involvement of the connector is suggested by the observation that some bacteriophage P22 connector mutations cause delay of maturation cleavage. The observed result is that P22 packages an oversized genome [74]. Unlike the T3/T7 maturation cleavage, the P22 maturation cleavage is not nucleotide sequence-specific. The packaging of an oversized genome by the P22 mutants is explained by assuming that the mutants have more rapid transmission of energy from ATPase ring to connector-associated peptide segments. That is to say, packaging continues further than it normally does, at the expense of cleavage.

In the case of bacteriophage T4, the above pathway for the terminase-catalyzed maturation cleavage explains the following otherwise puzzling observation. The small terminase protein, although not needed for the packaging motor, stimulates the ATPase activity of terminase and, thereby, inhibits premature (random) cleavage of a concatemer during packaging [8]. The blockage of premature cleavage is explained by the small terminase protein’s blocking of the alternative (DNA-cleaving) pathway for energy usage by removing the source of energy, the terminase-bound ATP. The small terminase protein also forms a multi-subunit ring that has a symmetry mismatch with the terminase, in the case of both T4 [8] and P22 [75].

Unlike the observation for bacteriophage λ [31], a drop in nanometry-observed, packaging-resisting force has not been observed at the later stages of bacteriophage ϕ29 *in vitro* packaging, in spite of the extensive nanometry-based studies of ϕ29, as referenced above. Bacteriophage ϕ29 also has a procapsid (equivalent to T3 capsid I) with a shell that is not smaller than the mature ϕ29 shell [58,76]. These two observations suggest that, in fact, ϕ29 does not have an operative type 2 cycle. In support, ϕ29 packages a monomeric DNA molecule, not a concatemer, *in vivo* and has a relatively short genome [6,40], thereby reducing the selection for mechanisms to accelerate packaging in the later stages. The crown region of the ϕ29 connector may have evolved to be relatively small because the primary function of the crown is either initiating or conducting the type 2 cycle. An outstanding question is whether or not ϕ29 is the product of reductive evolution from ancestors that had a complete terminase and, by inference, a type 2 cycle.

## 6. Comparison with thermal ratcheting in other systems

The feedback-controlled, ATP-dependent, DNA binding/releasing aspect of the mechanism proposed here is in the category of an ATP-dependent thermal ratchet. A thermal ratchet rectifies thermal motion and, as a general concept, includes miniaturized mechanical ratchets, as articulated in [77]. The sweeping-of-DNA aspect of the type 1 cycle proposed here is outside of the concept of a thermal ratchet, as are reach/bind/pull mechanisms for DNA packaging, one version of which has been called a “ratchet model” [78]. That is to say, some terminology is inconsistent.

Thermal ratcheting, as opposed to bind/pull, is not a new idea. Huxley [79] proposed an ATPdriven, bind/release thermal ratchet-based mechanism for muscle contraction a long time ago. Even earlier, Donnan ([80]; page 320) approached this concept in his discussion of the interaction of statistical mechanics and biology, but did not fully articulate it, because of the absence of knowledge of molecular biology. Currently, thermal ratchet-based cycles are in active consideration for actin/myosin and kinesin/microtubule motors (reviewed in [28,29,43,81,82]). Recent data indicate that thermal ratcheting of these eukaryotic motors is, as proposed here, part of a cycle that also has a non-ratcheting component [28].

In contrast to what occurs in non-viral eukaryotic motors, the bacteriophage DNA packaging ATPase does not perform the non-ratcheting component of the type 1 cycle proposed here. If this type 1 cycle is correct, an explanation is needed for separation of function to not only two different proteins, but also two different multimers. This explanation will ideally include an explanation for the symmetry mismatches among the components of the motor. Bacteriophages have had 1.6 billion years of evolution to match non-viral eukaryotic motors in incorporating these functions in one protein.

## 7. Symmetry mismatches, evolution and non-viral eukaryotic motor proteins

The type 1 cycle proposed here provides a new explanation for previously unexplained symmetry mismatches, including the connector/ATPase, 12/5 symmetry mismatch of the bacteriophage ϕ29 motor [58] and the symmetry mismatches of the T4 motor [8,83]. This explanation is that symmetry mismatching reduces thermal motion (noise)-derived signaling errors between the connector and ATPase multimers. Thus, evolutionary selection for symmetry mismatching occurs, even though the motor would work with a symmetry match that would simplify assembly of the motor.

This explanation has support from calculations that reveal the following in the case of communication networks. Although non-random events (signaling errors, in the case of the connector/ATPase multimer) can be generated from adding the thermal noise from two symmetry related sources, this occurrence of non-random events is suppressed by breaking the symmetry [84] and potentially yields greater signal amplification via stochastic resonance [84,85]. In the case of the proposed type 1 cycle, breaking the symmetry means having a symmetry mismatch between two multimers. By this reasoning, separating signal source (connector) and target (terminase/packaging ATPase) to separate multimers evolved to reduce thermal motion-induced signaling errors that occur in a single protein.

Symmetry mismatching to reduce thermal motion-derived signaling errors is a concept that extends to the long-unexplained symmetry mismatch between connector ring and outer shell of the capsid [5,6,76] and also to the symmetry mismatches in some chaperonin-protease complexes [86]. As shown directly for the connector during DNA packaging [23], facilitation of rotation [18] is not likely to be the reason for evolution of the mismatch in the case of the chaperonin-protease complexes either [87]. In the case of eukaryotic cellular (in contrast to viral) motors, the larger number of cooperating, non-symmetrically placed motors would, by the ideas presented here, reduce thermal motion-induced signaling errors enough to allow evolution of motor proteins with signal source and target in the same protein molecule.

Eukaryotic non-viral motors, such as kinesin/tubulin and myosin/actin motors, also (1) do not work against a predictably increasing load and (2) evolved long after bacteriophage DNA packaging motors. Thus, in the type 1 cycle proposed here, the relatively long distances for transmission of both information and energy are not in conflict with the finding of shorter transmission distances in eukaryotic cellular motors.

In support of the above interpretation of the symmetry mismatches, evidence exists that the ϕ29 connector/ATPase symmetry mismatch is not biophysically necessary for DNA packaging. An artificially generated, non-mismatched hexameric ATPase (and associated hexameric packaging RNA molecule) can be effective for *in vitro* DNA packaging [19,88]. Even assuming that the *in vitro* system used does not precisely mimic packaging *in vivo*, this observation means that ϕ29 could have evolved to make use of the assembly advantages of symmetry matching [89], unless something else promoted evolution toward symmetry mismatching. The motor mechanism proposed here predicts that this “something else” is suppression of signaling errors generated by thermal noise. A test of the proposed role of errors in the evolution of symmetry mismatching is the determining of how the frequency of errors (other than premature concatemer cleavage) depends on whether or not the ϕ29 motor is symmetry mismatched.

A pure thermal ratchet-based cycle is more primitive than the type 1 cycle proposed here, especially when ratchet-associated binding is chemically non-specific, as it is for clamping. Thus, the working assumption is that, whatever the most advanced bacteriophage DNA packaging motors are today, they started as thermal ratchets, perhaps without the packaging ATPase and with ATP-derived energy coming from only a type 2 or related cycle. In this case, the DNA would be packaged less tightly than has been described [4,90,91] for packaging ATPase-dependent bacteriophages such as ϕ29, λ, P22, T3, T4, T5 and T7. Perhaps, such low DNA density, packaging ATPase-less bacteriophages still exist in environmental niches that favor them.

## 8. Other, recently proposed type 1 cycles: Predictions of the cycle of Figures 2 and 3

At this point in time, the type 1 cycle proposed here appears to be alone in meeting all of the data-based constraints described in Section 3. Other proposed type 1 cycles include bind/pull cycles with all pulling dynamics occurring within the packaging ATPase multimer. One such proposed cycle is based on details of structure for the ϕ29 and T4 terminases [83], but (1) does not account for the subsequently obtained data of [52] in that it uses uncoordinated (though regulated) cleavage of ATP and (2) does not account for the data of [38] and [59] in that it does not incorporate the connector in the energetics. Moffitt *et al.* [52] have proposed a bind/pull mechanism with the pull generated by lock washer-like distortions of the relationships of at least four subunits within the ATPase ring (thereby explaining coordination), but again without considering the connector and introducing the concept that one of the 5 ATPase molecules in the multimer is different from the others. Yu *et al.* have more recently proposed a sterically driven push and roll mechanism whereby eccentric DNA motion assists the movement between ATPase molecules and ATP binding is delayed after four ATPase molecules have fired [92]. However, again, the connector is not incorporated in the motor.

Among the previous proposed type 1 cycles, one does have the connector and DNA packaging ATPase integrated in the motor. In this type 1 cycle, the connector initially blocks DNA motion from an ATPase-delivered power stroke; the connector later releases the DNA molecule in a burst [17,51]. This cycle basically has the roles of the connector and packaging ATPase inverted in relation to the type 1 cycle presented here. The proposed cycle of [17,51] presumably will be updated to explain the four minibursts subsequently revealed.

The type 1 cycle proposed here makes at least two predictions that are not made by bind/pull-based cycles and that can be tested. The first prediction is that the previously demonstrated dwells of the type 1 cycle are preceded by reverse motion of the DNA molecule. In fact, high-resolution nanometry of *in vitro* ϕ29 packaging does have an approximation of this pattern when packaging is dramatically slowed (to create a pause) by methylating the phosphates of a 10 base pair patch of DNA. In this case, forward movement interrupts the pause and is followed by several backward movements, each followed closely by forward movement (Figure 2 of [16]). One interpretation of the repeated backward/forward motion is the operation of the type 1 cycle with time scale stretched and forward motion inhibited by DNA methylation. That is to say, movements normally too rapid and small to resolve are made resolvable by the methylation. However, this potential interpretation of the data was not a focus of [16]. More probing of the interpretation of the repeated backward/forward motion is needed before the nanometry can be considered a test.

The second prediction is that the motions of the connector-associated, mobile peptide segments are both the signaling and DNA-driving aspects of the motor. This prediction can be tested by real time single motor fluorescence microscopy/nanometry of the DNA packaging process ([23], for example) with the various regions of the connector labeled with fluorescent probes and use of Förster resonance energy transfer, for example, to monitor packaging associated changes in peptide conformation.

In addition, the structural details of the type 1 cycle proposed here (or any proposed type 1 cycle) can tested by cryo-EM with 3-D reconstruction of DNA packaging motors with DNA still in the connector/ATPase channel. The cryo-EM will be dependent on the isolation and preliminary characterization of these various “motor intermediates” in a state as native as possible.
